# Variations in the Quality of Care at Large Public Hospitals in Beijing, China: A Condition-Based Outcome Approach

**DOI:** 10.1371/journal.pone.0138948

**Published:** 2015-10-02

**Authors:** Ye Xu, Yuanli Liu, Ting Shu, Wei Yang, Minghui Liang

**Affiliations:** 1 Department of Global Health and Population, Harvard School of Public Health, Boston, Massachusetts, United States of America; 2 National Institute for Hospital Administration, National Health and Family Planning Commission, Beijing, The People’s Republic of China; Sichuan University, CHINA

## Abstract

**Background:**

Public hospitals deliver over ninety percent of all outpatient and inpatient services in China. Their quality is graded into three levels (A, B, and C) largely based on structural resources, but empirical evidence on the quality of process and outcome of care is extremely scarce. As expectations for quality care rise with higher living standards and cost of care, such evidence is urgently needed and vital to improve care and to inform future health reforms.

**Methods:**

We compiled and analyzed a multicenter database of over 4 million inpatient discharge summary records to provide a comprehensive assessment of the level and variations in clinical outcomes of hospitalization at 39 tertiary hospitals in Beijing. We assessed six outcome measures of clinical quality: in-hospital mortality rates (RSMR) for AMI, stroke, pneumonia and CABG, post-procedural complication rate (RS-CR), and failure-to-rescue rate (RS-FTR). The measures were adjusted for pre-admission patient case-mix using indirect standardization method with hierarchical linear mixed models.

**Results:**

We found good overall quality with large variations by hospital and condition (mean/range, in %): RSMR-AMI: 6.23 (2.37–14.48), RSMR-stroke: 4.18 (3.58–4.44), RSMR-pneumonia: 7.78 (7.20–8.59), RSMR-CABG: 1.93 (1.55–2.23), RS-CR: 11.38 (9.9–12.88), and RS-FTR: 6.41 (5.17–7.58). Hospital grade was not significantly associated with any risk-adjusted outcome measures.

**Conclusions:**

Going to a higher grade public hospital does not always lead to better patient outcome because hospital grade only contains information about hospital structural resources. A hospital report card with some outcome measures of quality would provide valuable information to patients in choosing providers, and for regulators to identify gaps in health care quality. Reducing the variations in clinical practice and patient outcome should be a focus for policy makers in the next round of health sector reforms in China.

## Introduction

China has experienced surging demand for hospital care and rising expectations for high quality hospital services. The number of hospital admissions had more than doubled from 511 million in 2005 to 1,273 million in 2012. As the major service providers, 23,170 public hospitals provided 90% of total inpatient care [1]. However, patient satisfaction with the quality of hospital services is low and violence against physicians as a channel of expressing such dissatisfaction became more frequent [2, 3]. The perception of poor and varying quality of care is a root cause for an array of health sector issues including over-use of high level hospitals, under-use of lower level facilities, and deteriorating patient-doctor trust.

Quality of care needs improvement, and improvement starts with measurement. Currently China does not have a nationwide clinical quality performance information system, or a system of clinical quality indicators. Rigorous evaluation of the precise magnitude of problems has been very limited, as in many developing countries [4]. Such precise measurement requires good medical record keeping, openness to benchmarking and technical know-how. Traditional measures of hospital quality are heavily input-based structural indicators, which takes into account typically the number of beds, stock of equipment and the number of doctors and nurses in a hospital. Based on these variables, public hospitals are ranked into three grades (A is better than B, which is in turn better than C). Although the grade may provide a general sense of a hospital’s service capability, it is not a good measure of clinical quality because it overlooked the process and outcome of care. Nevertheless, higher grade public hospitals enjoy privileges of more public subsidy and higher service fees, and are allowed to provide more expensive drugs and higher-margin services. As a result, the grading system has had the unintended consequences of stimulating medical arm races.

This study proposes to use administrative data to derive hospital report cards with condition-based outcome measures of health care quality. The outcome approach has the advantage of measuring the direct impact of health services on patients’ health status. It urges providers to focus on the “ends” of health care while allowing autonomy and flexibility of the “means” to achieve the ends. A key task is to properly adjust for differences in patient case mix and health risks among hospitals. To this end we carried out careful statistical analysis with the detailed patient’s demographic and clinical information in the administrative data.

## Materials and Methods

### Hospital and Data

We undertook a retrospective analysis using routinely collected inpatient discharge summary records, which contained over 100 variables including the patient’s socio-demographic characteristics (e.g. age, sex, race/ethnicity, and insurance status), up to six diagnoses, treatment records, and service charges in sub-categories. The inpatient discharge summary records follow a national template and have standardized disease coding in ICD–10. Confidential information like name and residential address were excluded from the database.

The hospital sample in this study were 39 Level–3 non-military public hospitals in Beijing, including 26 general hospitals, 9 traditional Chinese medicine hospitals, and 4 specialty hospitals (1 cardiovascular hospital, 2 cancer hospitals and 1 chest hospital). All patients admitted to the study hospitals during 2005 and 2010 (N = 4,216,275) were initially included. All data were collected through the Beijing Public Health Information Center, an online secured data platform operated by a third-party organization contracted with the Beijing Municipal Health Bureau.

The quality of the data in Beijing is considered the best in China, especially since Beijing had strengthened medical recording during its pilot program to design a DRG payment system since 2005 by providing additional training on record-keeping standards, standardizing reporting practice and switching to electronic reporting. Any record that has three missing required items is classified as “secondary” and will lead to deduction of points in the annual hospital examination. In addition, a random sample of 30% of inpatient discharge summaries is validated against charts by the collection agency.

### Outcome Quality Measures

Six outcome quality measures were chosen. Four are risk-standardized mortality rates (RSMRs), for which we chose acute myocardial infarction (AMI), stroke, pneumonia, and coronary artery bypass graft (CABG) as tracer conditions. The other two are risk-standardized post-procedural complication rate (RSCR) and failure-to-rescue rate (FTR) based on the occurrence of 20 major post-procedural complications. Failure-to-rescue rate characterizes the likelihood of deaths as an outcome among patients who developed post-procedural complications. The literature suggests that mortality rates, complication rate and failure-to-rescue rate measure different aspects of quality, and it may be helpful to include all three to provide a more comprehensive depiction of clinical quality [5–8] Tracer conditions and complications were identified using ICD–10 codes; procedures were identified using ICD-9-CM3 codes (S1 Table).

### Statistical Analysis

The central statistical strategy for developing comparable outcome quality measures was to adjust for hospital case mix and patient severity. Most risk-adjustment approaches characterize the variance in patient outcome as sourcing from the patient, hospital and random variations, and use an indirect standardization technique to produce standardized performance measures [9]. This is done in two steps. First, using a hierarchical mixed linear model (HLM), we modeled the log-odds of the outcome of interest (e.g. mortality) as a function of patient risk factors including patient demographic variables (age and sex), indicators of health and severity of condition (prior hospitalization, condition at admission, and comorbidities), year dummies (2006–2010) and a hospital-specific random intercept, leaving the residual variance a function of hospital characteristics and random variation. Compared with standard logistic regression (SLR), the HLM takes into account data clustering at the hospital level. It is common to use SLR as a precursor to HLM for selecting relevant predictive covariates and this was our approach too [10]. For the sake of simplicity, we only report the results from the HLM models. In the second step, we applied the indirect standardization to derive risk-standardized outcome measures for each hospital (see S1 File for details of statistical methods). The outcome quality measures were calculated as the ratio of the sum of expected number of outcomes at a hospital conditioned on its patient-mix and hospital-specific intercept, divided by the sum of expected number of outcomes at a counterfactual hospital with the same patient case-mix given the overall intercept (overall mean).

We restricted selection of patients to adults over 18 year-old and under 90 year-old, and categorized age into five groups (18–45, 46–55, 56–65, 66–75, 76–90). Prior hospitalization indicates whether the patient was admitted to a hospital in the previous year regardless of condition. Patients may be admitted in an emergency, urgent or regular condition, which is used as a proxy for the severity level. Comorbidities were identified from secondary diagnosis codes using the Elixhauser’s method [11, 12] Confusion of comorbidities with complications was minimized by consulting a physician regarding the relative likelihood of a secondary code as comorbidity or complication for specific conditions and excluding highly-likely confusions, and ensuring no overlapping in the definition of comorbidities and complications for any indicator condition.

The HLM analyses were performed using the PROC GLIMMIX with SAS 9.2. The procedure applied a restricted (residual) pseudo-likelihood algorithm to get maximum likelihood estimates. Standard errors were calculated using empirical bootstrapping [10]. For each model, we directly re-sampled 1000 times with repeated hospital-level sampling and re-fit the hierarchical model with each sample, generating the confidence intervals for the estimated quality measures.

After we developed the hospital-specific risk-standardized outcome quality measures, we further examined the correlations between these quality measures and hospital grade.

### Ethics Statement

This study was approved by the Office of Human Research Administration at Harvard Longwood Medical Area. Patient records/information was anonymized and de-identified prior to analysis, so no written consent was obtained. The authors have declared that no competing interests exist.

## Results

We analyzed 48,678 AMI patients, 112,392 stroke patients, 36,726 pneumonia patients, 26,226 CABG patients, and 1,784,989 patients who developed defined post-procedural complications among which 201,905 patients eventually died. Between 2006 and 2010, the increases in the volume of patients admitted for AMI, CABG, pneumonia and stroke were 61.2%, 66.7%, 47.5% and 25.7%, respectively. The crude mortality rates (CMRs) of AMI, stroke and CABG declined slightly (from 6.86% to 5.39%, 4.69% to 3.75%, and 2.56% to 1.61%, respectively); but CMR-pneumonia increased slightly (from 6.68% to 7.97%). The crude post-procedural complication rate increased from 6.59% to 14.14%, whereas the crude failure-to-rescue rate decreased from 7.08% to 6.11%.

All HLM models met the convergence criterion and fit statistics showed no evidence of over-dispersion. Model results were presented in Table 1. As expected, older age and emergency admission were associated with worse outcome. Patients without prior hospitalization had smaller odds of adverse outcome (deaths or complications). Consistent with the literature, male tended to have worse outcome than female, except for AMI patients [13–15]. The intra-class correlation (ICC) was 0.192 for the AMI model, suggesting that 19.2% of the variation can be attributed to the hospital level random effect. ICC was 0.183 for the stroke model, 0.273 for the pneumonia model, 0.217 for the CABG model, 0.379 for the complication rate model, and 0.216 for the FTR model.

**Table 1:** Risk-standardization models (HLM) results.

Outcome measure	Age46-55	Age56-65	Age66-75	Age76+	Male	Emergency admission	Urgent admission	First Admission	No. of Sig. comorbi-dities	C statistic	Max Re-scaled R^2^
RSMR-AMI	0.04 (0.02, 0.06)	0.10 (0.08, 0.13)	0.18 (0.15, 0.21)	0.50 (0.46, 0.55)	0.73 (0.67, 0.79)	2.03 (1.81, 2.28)	0.78 (0.71, 0.86)	0.61 (0.56, 0.66)	10	0.83	0.24
RSMR-Stroke	0.47 (0.40, 0.56)	0.54 (0.48, 0.60)	0.48 (0.43, 0.53)	0.65 (0.61, 0.71)	n.s.	17.26 (15.76, 18.91)	2.29 (2.13, 2.46)	n.s.	22	0.81	0.21
RSMR-Pneumonia	0.09 (0.06, 0.11)	0.17 (0.13, 0.22)	0.30 (0.24, 0.35)	0.57 (0.51, 0.63)	1.20 (1.10, 1.32)	9.65 (8.42, 11.05)	1.91 (1.75, 2.09)	0.80 (0.73, 0.88)	24	0.83	0.25
RSMR-CABG	0.32 (0.18, 0.55)	0.20 (0.14, 0.29)	0.24 (0.18, 0.32)	0.49 (0.38, 0.62)	0.72 (0.59, 0.87)	4.16 (3.07, 5.63)	1.27 (1.05, 1.55)	0.74 (0.58, 0.94)	5	0.70	0.08
RS-CR	0.22 (0.21, 0.23)	0.33 (0.33, 0.34)	0.45 (0.43, 0.46)	0.64 (0.63, 0.65)	1.20 (1.19, 1.22)	4.73 (4.55, 4.92)	2.06 (2.03, 2.09)	0.77 (0.76, 0.78)	26	0.91	0.57
RS-FTR	0.22 (0.20, 0.24)	0.33 (0.31, 0.35)	0.37 (0.35, 0.40)	0.58 (0.55, 0.60)	1.05 (1.01, 1.09)	6.74 (6.35, 7.16)	1.89 (1.81, 1.97)	0.73 (0.71, 0.76)	17	0.80	0.2

Note: Authors’ calculations. 95% confidence interval for parameter estimates in parentheses. n.s. means non-significant

The analysis produced four notable findings. First, the risk adjustment method served well in reducing random variations when hospital case number is small, in other words, in the low information context. The numeric range of the crude outcome measures were generally wide and differed by condition, and the zero outcomes posed difficulty for interpretation in some cases. For example, CMR-AMI ranged from 1.35% to 100%, whereas the CMR-CABG ranged from 0 to 9.09%. The risk adjustment method stabilized the outcome measures so that they had narrower ranges and smaller dispersion, which in theory represented the true systematic variations in quality (Table 2). Risk adjustment also eliminated variations due to different patient case mix among hospitals. As a result, the relative ranking of hospitals by risk-adjusted outcome measures is substantially different from that by the crude measures.

**Table 2:** Contrast of the range of values of hospital clinical quality measures between crude and risk-standardized measures.

Outcome measure	Crude Rate (%)	RSMR-HLM (%)
AMI mortality	1.35–100	2.37–14.48
Stroke mortality	0–75	3.58–4.44
Pneumonia mortality	0–24.37	7.2–8.59
CABG mortality	0–9.09	1.55–2.2
In-hospital Complications	0–46.47	9.90–12.88
Failure-to-rescue	0.99–25.95	5.17–7.58

Note: Authors’ calculations. HLM refers to risk-standardized results from the hierarchical mixed linear models.

**Fig 1:**
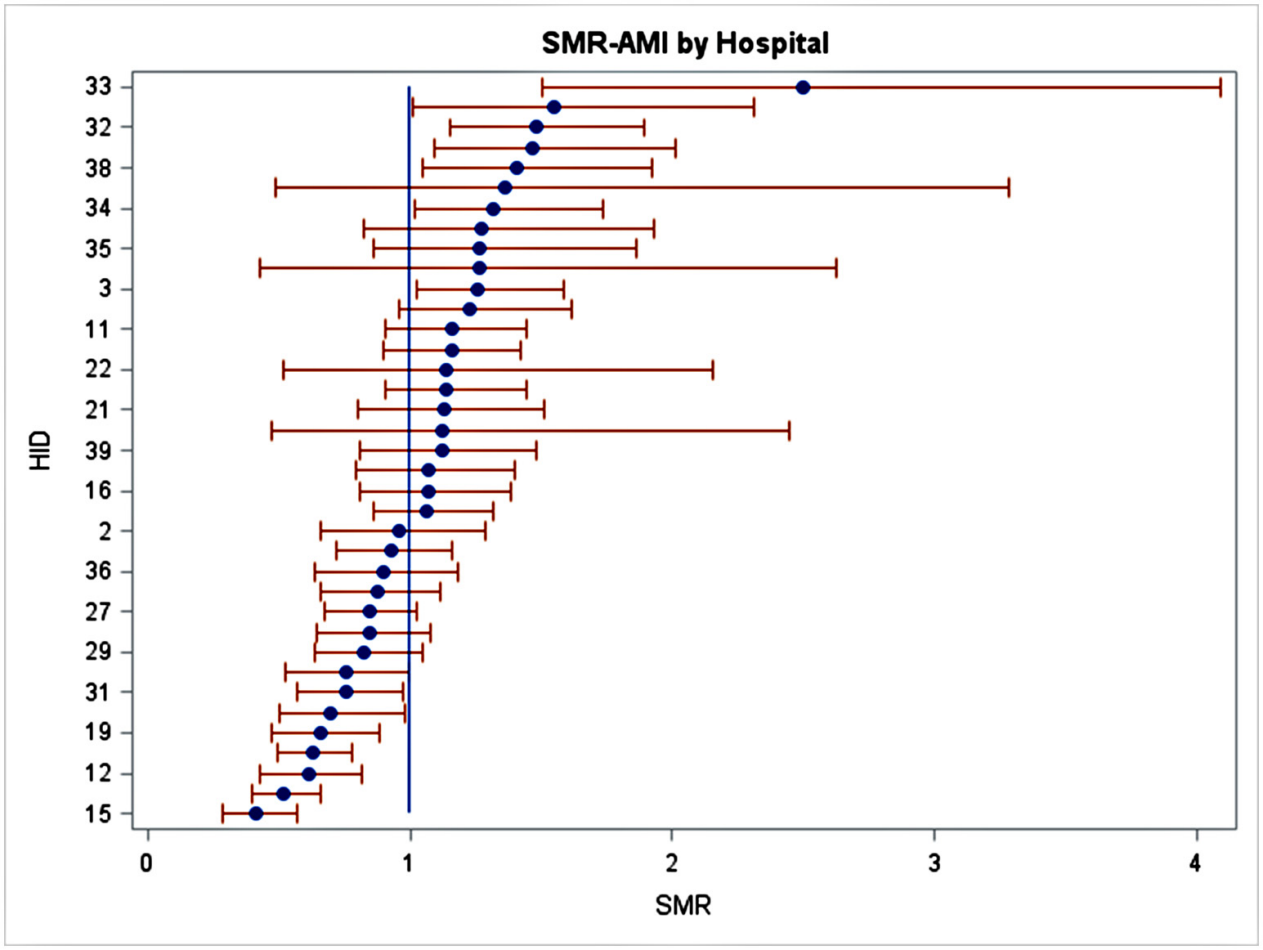
Observed mortality-to-expected mortality ratios for AMI patients at study hospitals. Source: Author’s calculations. Note: Y axis: hospital ID, X axis: standardized mortality ratio (SMR). SMR>1 indicates higher mortality than expected, thus worse clinical outcome.

Second, the variations in hospital clinical quality were still substantial after risk-standardization (S1 Fig). As an example, Fig 1 shows the standardized mortality rate (SMRs) of AMI patients from 37 hospitals that treated at least 30 AMI patients. If a hospital’s SMR is not significantly different from one, then the hospital is performing as good as expected; if a hospital’s SMR is significantly smaller than one, the hospital’s performance is better than expected, and vice versa. The greater the SMR deviates from one, the more extreme quality of a hospital. Hospitals can then be ranked by how far they are from their expected performance level. The number of hospitals that had higher-than-expected outcomes (thus worse quality) was 7 on RSMR-AMI (18.9%), 4 on RSMR-Stroke (10.2%), 7 on RSMR-pneumonia (17.9%), 3 on RSMR-CABG (17.6%), and 12 on RSCR and FTR (30.8%). We observed the largest gap between the top hospital and the bottom hospital for AMI treatment, in which the high quality hospital had 60% less mortality than would-be-expected and the low quality hospital had 150% more mortality than would-be-expected. This gap ranged 18% to 35% for the other measures.

Third, there was low correlation among the risk standardized outcome measures. No hospital performed better-than or worse-than-expected on all six measures, although having more measures better than expected may suggest a general strength in a hospital’s clinical quality. The only significant correlations observed are between RSMR-AMI and FTR (ρ = 0.45, p = 0.02); between RSMR-CABG and FTR (ρ = 0.31, p = 0.04) and between RSCR and FTR (ρ = -0.50, p<0.001). The results suggested that a condition-based approach to outcome quality measures may be more appropriate than a global measure of outcome quality, i.e. hospital-wide mortality rate. The mechanisms underlying the observed significant association warrants further qualitative investigation.

Finally, we contrasted the risk-standardized outcome measures between the 27 Grade A and 12 Grade B hospitals in the study sample. We did not find that Grade-A hospitals performed significantly better than Grade B hospital on any risk-standardized outcome measures (Table 3).

**Table 3:** Associations between hospital grade and risk-standardized outcome measures.

	RSMR-AMI	RSMR-stroke	RSMR-pneumonia	RSMR-CABG	RSCR	FTR
Grade A (N = 27)	5.86%	4.20%	7.78%	1.96%	11.27%	6.43%
Grade B (N = 12)	7.01%	4.13%	7.79%	1.87%	11.64%	6.36%
p-value	0.13	0.21	0.85	0.31	0.2	0.72

Note: Authors’ calculations. Results are from ANOVA tests.

## Discussion

Public hospitals are the backbone of the health care delivery system in China. In recent years, demand for public hospitals service has increased rapidly due to demographic and epidemiological transitions, higher income, and expansion of social health insurance. It is critical that the public hospitals are able to respond to this surging demand and consistently provide standardized, high-quality care that improve patient outcome. However, without a scientific and transparent performance information system, improvement of health care quality can be a moving target.

We aimed to assess the clinical quality of Level–3 public hospitals in Beijing with a set of refined outcome measures based on tracer conditions and adjusted for patient risk-profiles at admission. The measures we used provided more comparable and reliable metrics for comparing the relative clinical quality of inpatient services than crude measures. The results revealed substantial quality variations among these hospitals, which should draw policy attention. 11%-31% of hospitals performed worse than what would be expected on specific outcome measures, and more hospitals performed worse-than-expected rather than better-than-expected in general. Further studies should aim at understanding the success factors of high performance outliers. Hospital management, especially targeted quality improvement initiatives may play an important role.

An important conclusion from this study is that the structural input-based grading system is no longer relevant for comparing the quality of care among large public hospitals. Most of our study hospitals are equipped with the latest technology and able staff. Under the current policy, higher grade public hospitals will continue to receive greater government subsidies for expansion. This financial reward may be misplaced as our results showed no correlation between high hospital grade and better patient outcome. The result confirmed that adding the outcome dimension to quality assessment is necessary and important.

Finally, these results may provide some sense of the quality of care at the study hospitals when benchmarked internationally. A few studies have noted the general declining trend of hospital mortality as a sign of improved quality of care. [16] The outcome quality of the study hospitals seemed to be on par with the average level of hospitals in industrial countries. For example, the literature has documented risk-standardized hospital mortality rate for AMI in the range of 5.1%-19.3% in the U.S. in 2004–2007 [17, 18], 7.0% -21.7% in Norway in 1997–2001 [19], 15% on average in The Netherlands in 2009 [20], and 7.8% in Japan in 2008 [21]. In-hospital mortality of stroke patients ranged from 2.3%-13.7% in the U.S.[17], 6.9%–21.6% in Norway [19], and 5.4% on average in Berlin [22]. For pneumonia, the literature documented 4.5% to 28.4% in-hospital mortality in the U.S. [17, 18] Considering that Beijing represents the most resource-rich region in China and the study hospitals are among the best in the country, the results are probably not surprising. A cautionary note is that the numbers are not directly comparable due to differences in the population studied and the exact way the measures were derived.

In sum, we argue that a more rigorous approach to assess hospital quality, perhaps a report card with outcome measures, should be adopted by Chinese policy makers and such information should be made public. International experience suggested that the publication of hospital performance data provides added values to help patients make informed choices of care providers, as well as increase peer pressure for quality improvement [23, 24]. If the Chinese government decides to make public hospital performance report cards available to the public, this study provide value-added to the methodology to derive outcome quality measures and could serve as a basis for developing a comparable clinical quality performance information system nationwide.

However, the current policy environment in China does not provide sufficient motivations for quality improvement. The health administrative departments implement loose regulation on poor quality because the public hospitals are their main political constituents. Hospital payment and physician income are not associated with quality of care. Only a few hospitals voluntarily take on quality improvement initiatives. This is likely to change rapidly. Acknowledging the importance of the quality of health care, especially of the public hospital sector, the Chinese government has included public hospital reforms as one of the priorities of health system reforms in the 12th Five-Year Strategic Development Plan (2011–2015) [25]. A new Center for Medical Service Management has been established in January, 2015 to provide guidance and to implement interventions for appropriate care and quality improvement [26]. It is clear that more reliable, consistent and higher quality of care is essential for meeting patient expectations and instrumental for pushing forward other policy reforms.

This study has limitations. Our use of outcome measures did not avoid the inherent limitation that the causal link between clinical quality and immediate patient outcome may be uncertain. Patient outcomes may be affected by non-clinical factors like compliance and participation, appropriate discharge management and even social support. These factors need to be adjusted for in assessing the outcome quality, but in practice this is often complicated by the cost and feasibility of data collection. We were not able to evaluate and report 30-day outcomes or re-admissions because the patient follow-up information is not available. Another limitation is the generalizability of our method and findings to other hospitals in China. There are challenges related to the quality of such routine administrative data in other localities and hospitals, for example, lower level hospitals do not report inpatient discharge record data to the health administrative departments, but quality concern may be greater in these facilities. Improving the availability and comprehensiveness of data for quality assessment and monitoring should be a priority nation-wide.

This study contributes to the limited literature that has empirically documented the overall level and variation of the clinical quality of healthcare services in developing countries. Thousands of lives could be saved if continuous quality improvement is implemented at these hospitals with evidence-based and proven methods. Adopting a quality monitoring system could facilitate this progress, and may also serve as a foundation for pay-for-performance reforms in the future.

## Supporting Information

S1 Fig Observed outcome-to-expected outcome ratios for six tracer conditions at study hospitals. Source: Author’s calculations. Note: Y axis: hospital ID, X axis: standardized mortality ratio (SMR). SMR>1 indicates higher mortality than expected, thus worse clinical outcome.
(PDF)

S1 File Details of risk adjustment methods. (PDF)

S2 File Minimal data set underlying the study findings. (PDF)

S1 Table Table on inclusion and exclusion criteria for case selection. (PDF)
