# Supplementary material for: Variations in the Quality of Care at Large Public Hospitals in Beijing, China: A Condition-Based Outcome Approach
Source: PLoS One. 2015 Oct 2;10(10):e0138948. doi: 10.1371/journal.pone.0138948 (PMC4592271; doi:10.1371/journal.pone.0138948)
Supplement: S1 Fig — Source: Author’s calculations. Note: Y axis: hospital ID, X axis: standardized mortality ratio (SMR). SMR>1 indicates higher mortality than expected, thus worse clinical outcome. (PDF) [file pone.0138948.s001.pdf]

S1_Figures

Figure A. Observed mortality-to-expected mortality ratios for AMI patients at study hospitals

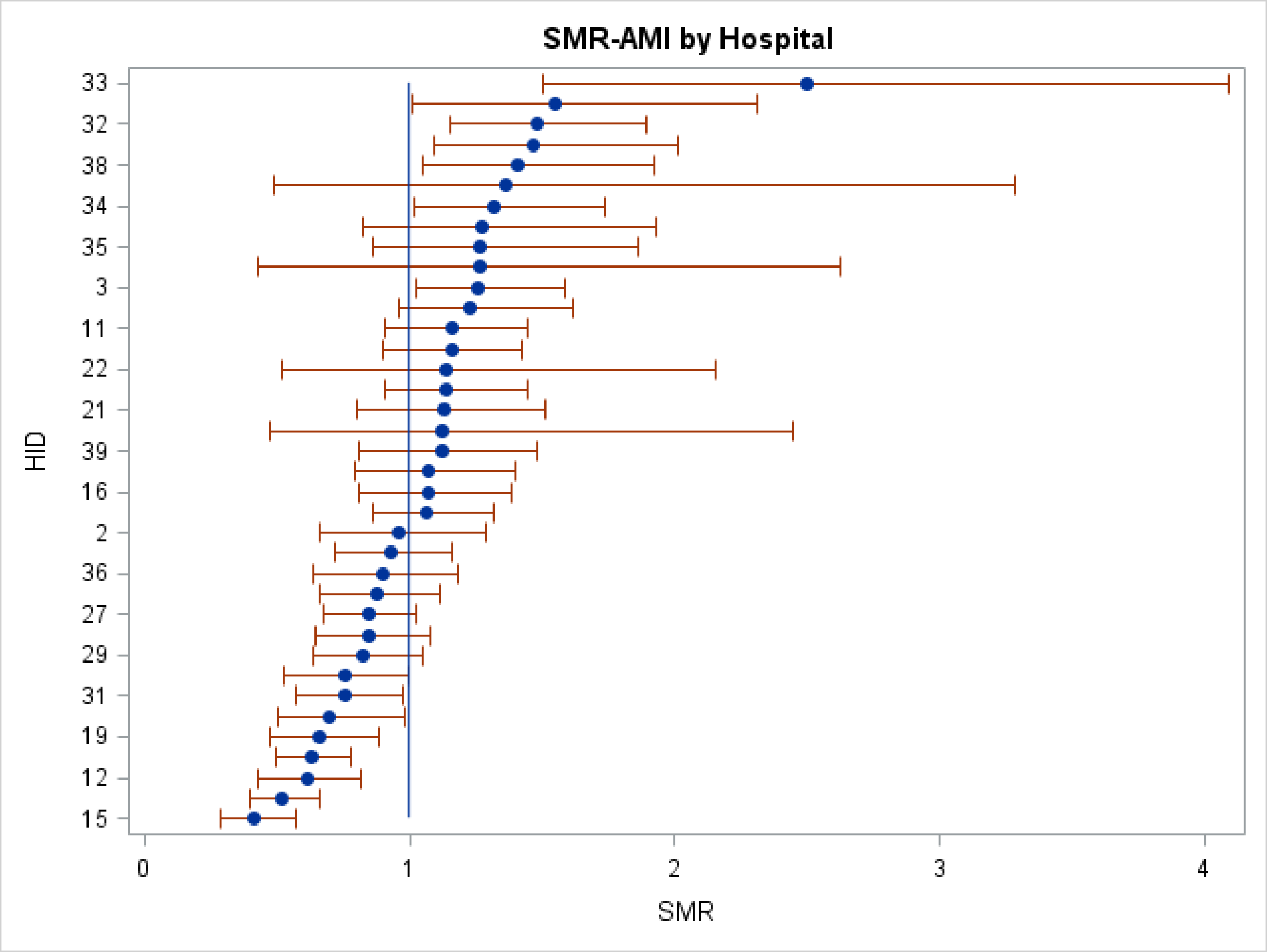

Note: Authors' calculation. Y axis: hospital ID, X axis: standardized mortality ratio (SMR). SMR>1 indicates higher mortality than expected, thus worse clinical outcome.

Figure B. Observed mortality-to-expected mortality ratios for stroke patients at study hospitals

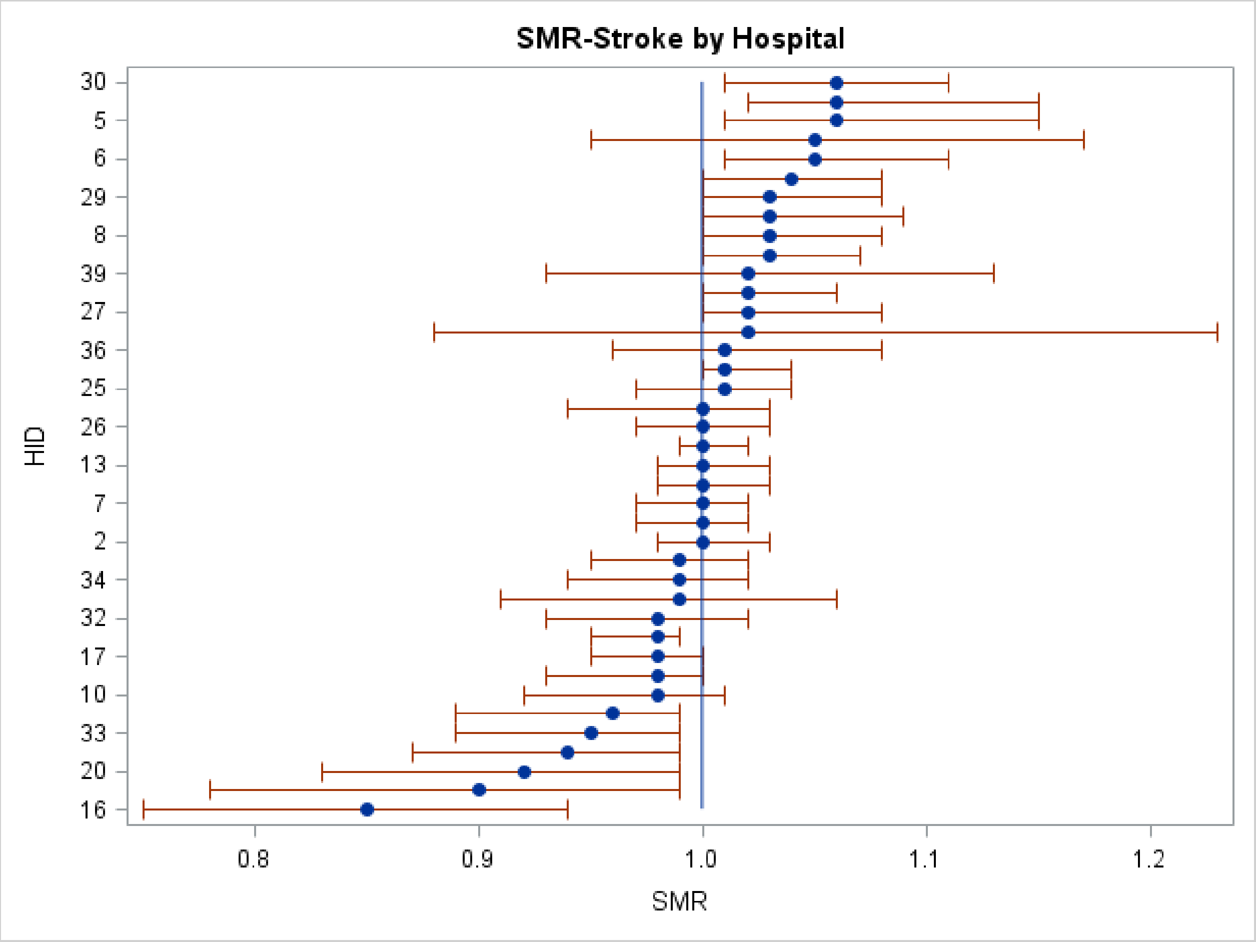

Note: Authors' calculation. Y axis: hospital ID, X axis: standardized mortality ratio (SMR). SMR>1 indicates higher mortality than expected, thus worse clinical outcome.

Figure C. Observed mortality-to-expected mortality ratios for pneumonia patients at study hospitals

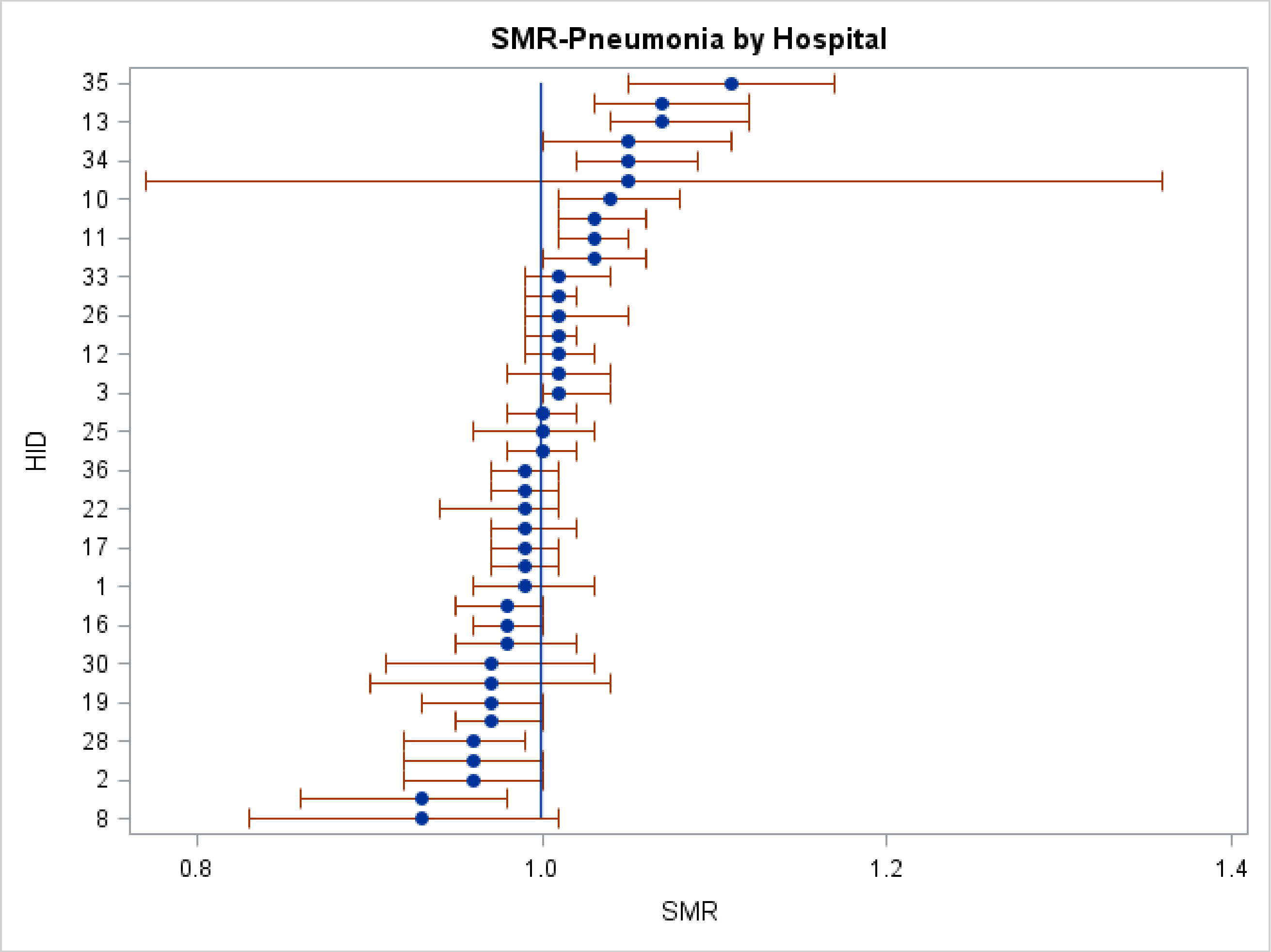

Note: Authors' calculation. Y axis: hospital ID, X axis: standardized mortality ratio (SMR). SMR>1 indicates higher mortality than expected, thus worse clinical outcome.

Figure D. Observed mortality-to-expected mortality ratios for CABG patients at study hospitals

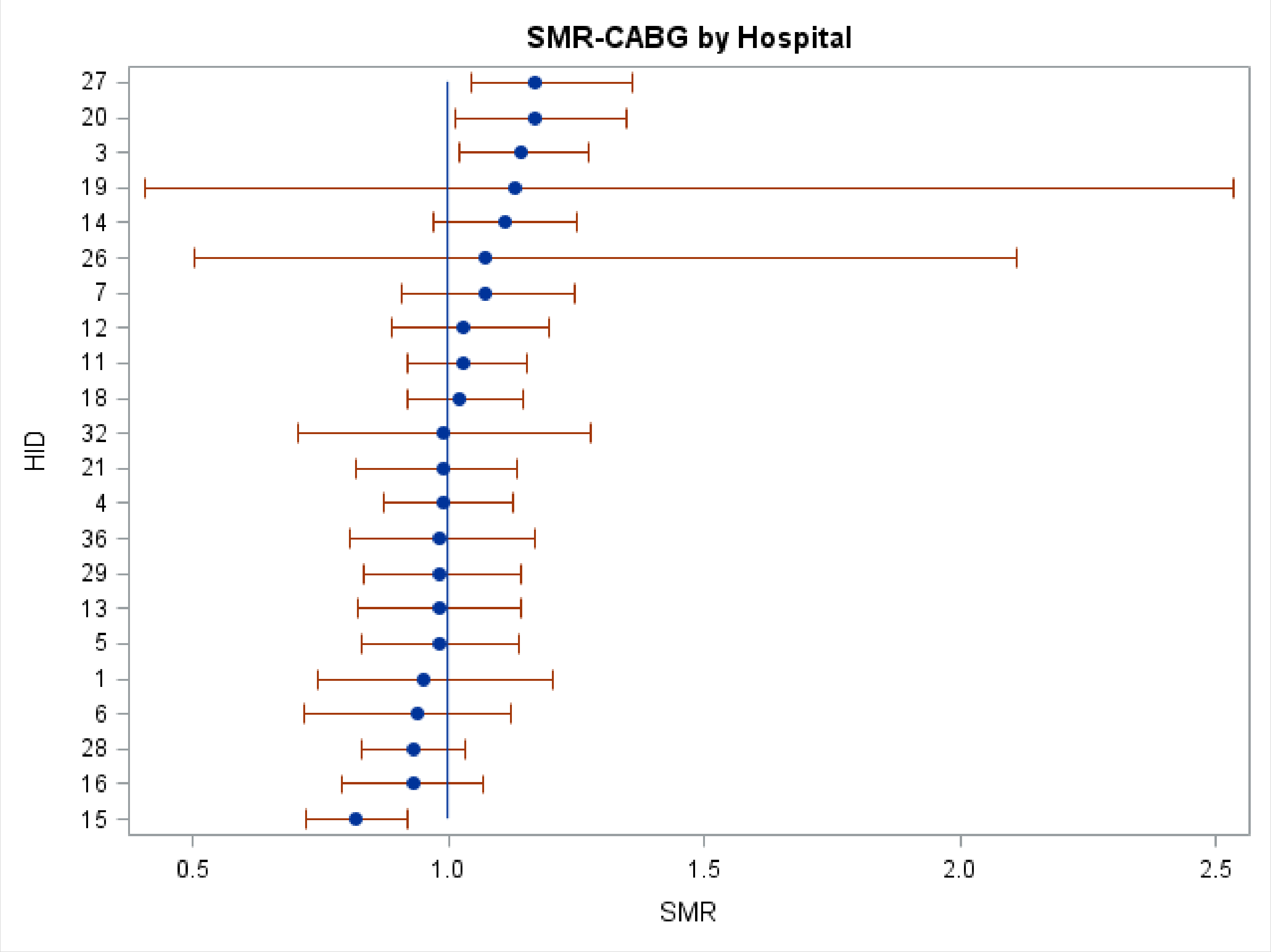

Note: Authors' calculation. Y axis: hospital ID, X axis: standardized mortality ratio (SMR). SMR>1 indicates higher mortality than expected, thus worse clinical outcome.

Figure E. Observed mortality-to-expected ratios of post-procedural complications at study hospitals

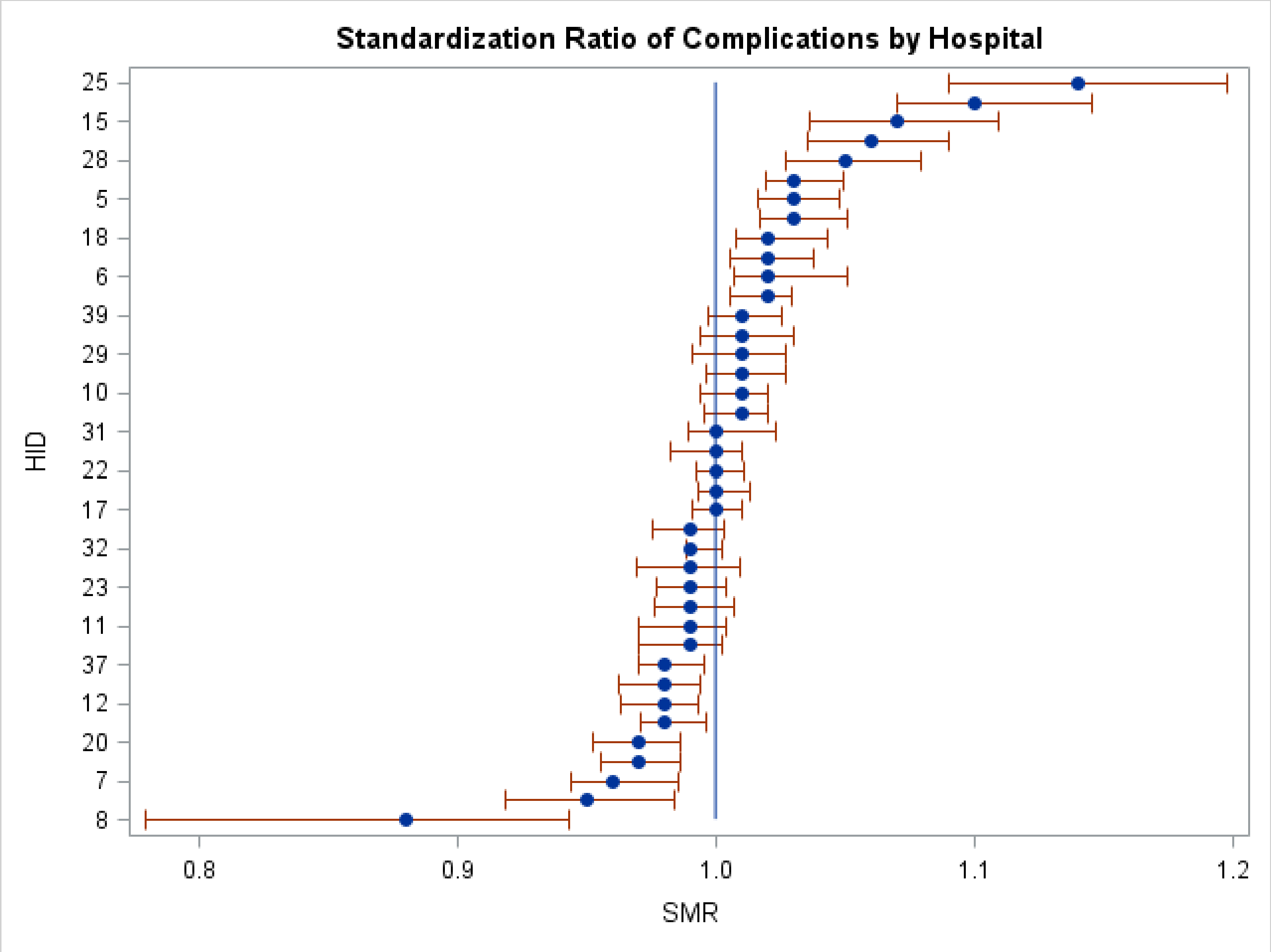

Note: Authors' calculation. Y axis: hospital ID, X axis: standardized ratio of complication (noted as SMR for complications). SMR>1 indicates higher mortality than expected, thus worse clinical outcome.

Figure F. Observed mortality-to-expected ratio of failure-to-rescue at study hospitals

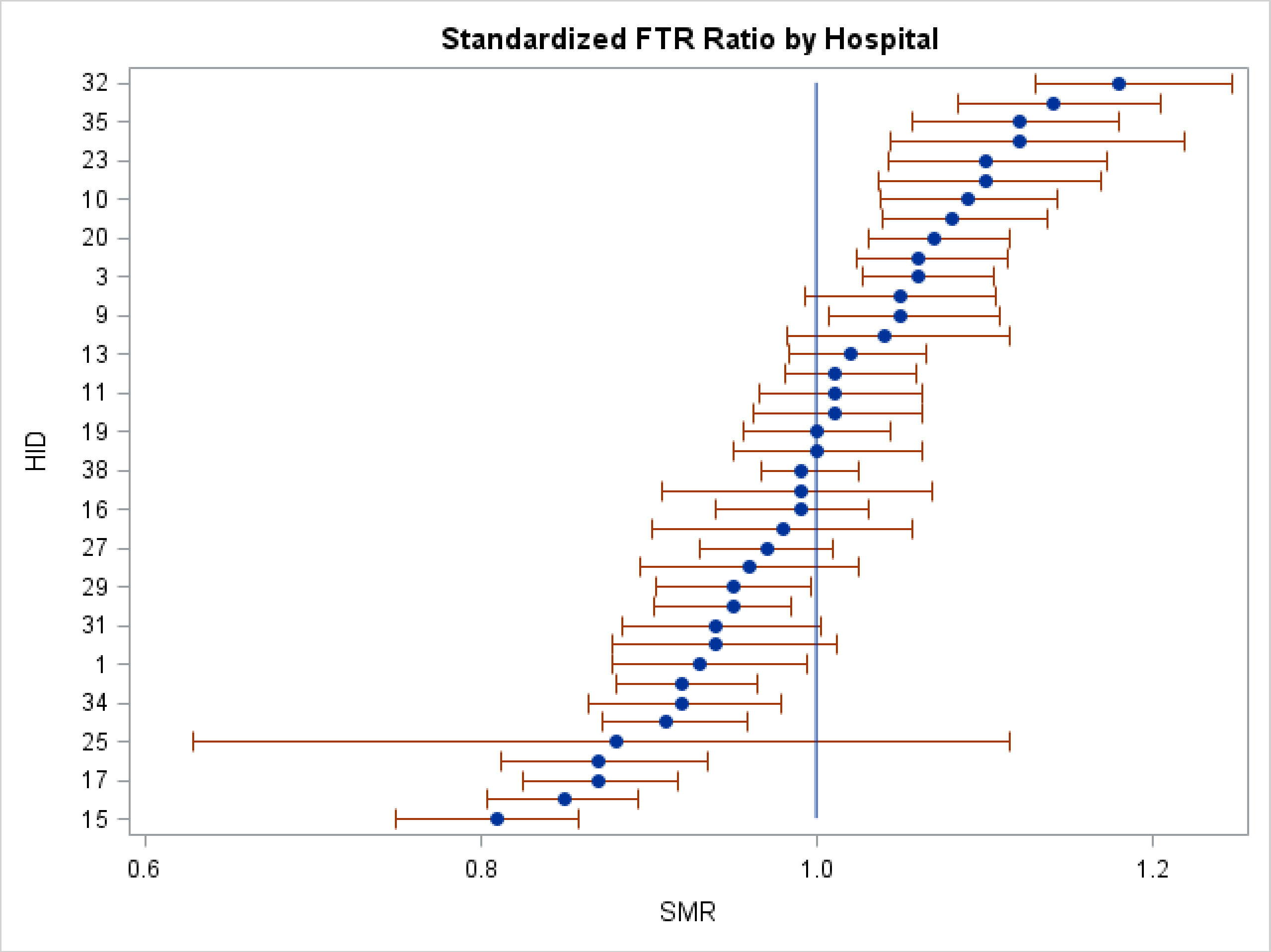

Note: Authors' calculation. Y axis: hospital ID, X axis: standardized ratio of failure-to-rescue (noted as SMR for FTR). SMR>1 indicates higher mortality than expected, thus worse clinical outcome.
