# Supplementary material for: Variations in the Quality of Care at Large Public Hospitals in Beijing, China: A Condition-Based Outcome Approach
Source: PLoS One. 2015 Oct 2;10(10):e0138948. doi: 10.1371/journal.pone.0138948 (PMC4592271; doi:10.1371/journal.pone.0138948)
Supplement: S1 File — (PDF) [file pone.0138948.s002.pdf]

S1_File

Risk-adjustment methods

In general terms, the indirect risk standardization involves two steps:

Step One: Establish the risk-adjustment model: Yij ~ f (patient characteristics), where Yij is the interested outcome for patient i from hospital j. Derive the predicted value of Y for each individual ($\widehat{\boldsymbol{Yij}}$)).

Step Two: Calculate the risk-standardized outcome measures for each hospital by: ($\sum_{i=1}^{n} \boldsymbol{Observed\ outcomes} / \sum_{i=1}^{n} \boldsymbol{Expected\ outcome}$) * population average outcome rate.

Standard Logistic Regression:

$Y_{ij} \sim$ Bern (1, π_{ij})

logit (π_{ij}) = $\alpha + \beta X_{ij} + \mathcal{E}_{ij}$ (1)

Hierarchical Logistic Model:

$Y_{ij} \sim$ Bern (1, π_{ij})

logit (π_{ij}) = $\alpha_{0j} + \beta X_{ij} + \mathcal{E}_{ij}$ (2)

$\alpha_{0j} = \alpha + \mu_j$

Where i = 1, …, i is the patient level indicator, j = 1,….j is the hospital level indicator, Y is the outcome of the patient (death/complication=1, survival=0), π_{ij} is the probability of death for patient i in hospital j, conditional on patient-level risk factors x_{ij}. The random effect model expresses that the logit is the sum of hospital-specific intercept αj and effects of patient-specific effects βX_{ij}, while the hospital intercept is a random variable with mean α and random variation $\mu_j \sim N(0, \tau^2)$.

In the standard logistic regression models, the SMR is calculated as:

$$\sum_{j=1}^{ni} Yij \,/\, \sum_{j=1}^{ni} E(Yij|\alpha, \beta, Xij); \quad (3)$$

in the hierarchical logistic regression models, the SMR is calculated as:

$$\sum_{j=1}^{ni} E(Yij|\alpha j; Xij, \alpha, \beta, \tau^2) \,/\, \sum_{j=1}^{ni} E(Yij|\alpha, \beta, \tau^2) \quad (4)$$
