# Supplementary material for: Variations in the Quality of Care at Large Public Hospitals in Beijing, China: A Condition-Based Outcome Approach
Source: PLoS One. 2015 Oct 2;10(10):e0138948. doi: 10.1371/journal.pone.0138948 (PMC4592271; doi:10.1371/journal.pone.0138948)
Supplement: S2 File — (PDF) [file pone.0138948.s003.pdf]

S2_File

Minimal Data Sample Codebook

Variable Name	Value
sjnf	Year (2006-2010)
jgdm	Hospital code
S0101	Payment method
S0102	Number of hospitalization
S0104	Sex (1=male; 2=female)
S0105	Age
S0301	Discharge date
S0401	Condition at admission (1=emergency; 2=urgent; 3=elective)
S0402	Diagnosis at admission (ICD-10)
S0501	Primary diagnosis at discharge (ICD-10)
S050101	Outcome of treatment for primary diagnosis (1=recovered; 2=relieved symptoms; 3=no change; 4=died; 5=others)
S0502	Secondary diagnosis at discharge (ICD-10)
S050201	Outcome of treatment for secondary diagnosis

S0506	other diagnosis at discharge (ICD-10)
S050601	Outcome of treatment for other diagnosis
S0507	other diagnosis at discharge (ICD-10)
S050701	Outcome of treatment for other diagnosis
S0508	other diagnosis at discharge (ICD-10)
S050801	Outcome of treatment for other diagnosis
S0509	other diagnosis at discharge (ICD-10)
S050901	Outcome of treatment for other diagnosis
S0510	other diagnosis at discharge (ICD-10)
S051001	Outcome of treatment for other diagnosis
S0511	other diagnosis at discharge (ICD-10)
S051101	Outcome of treatment for other diagnosis
S0503	In-hospital complication (ICD-10)
S0504	External causes of injury and poisoning (ICD-10)
S0505	Surgery code 1 (ICD-9-CM3)
S0512	Surgery code 2 (ICD-9-CM3)
S0513	Surgery code 3 (ICD-9-CM3)

S0514	Surgery code 4 (ICD-9-CM3)
S0515	Surgery code 5 (ICD-9-CM3)
N0601	Total hospitalization expense

Sample data

SJNF	JGDM	SOL01	S0102	S0104	S0105	S0201	S0301	S0401	S0402	S0501	S050101	S0502	S050201
2,006	PDY000570	3	1	1	54	24-Apr-2006	##########	2	R06.003	I21.202	2	I25.105	2
2,006	400005599	1	3	1	67	##########	##########	2	R07.401	I21.402	1	I25.101	2
2,006	400686312	4	1	2	60	07-Apr-2006	19-Apr-2006	2	I21.101	I21.101	2	I25.101	2
2,006	400688545	4	1	1	46	22-Feb-2006	24-Feb-2006	2	I21.9	I21.9	2	I25.101	2
2,006	400014014	4	1	1	73	##########	26-Oct-2006	2	I21.009	I21.009	2	I25.103	2
2,006	400688385	1	1	1	90	19-Oct-2006	19-Oct-2006	1	I21.304	I21.005	4	I50.902	4
2,006	400003198	4	1	1	48	##########	##########	1	I21.902	I21.002	5		
2,007	400686312	4	1	1	79	14-Jan-2007	01-Feb-2007	2	I21.303	I21.303	2	I25.101	2
2,007	400686312	1	2	1	75	11-Apr-2007	18-Apr-2007	2	I21.404	I21.404	2	I25.105	2
2,007	400686312	1	1	1	67	02-Nov-2007	17-Nov-2007	1	I21.403	I21.403	2	I25.105	2
2,007	400688545	1	2	1	49	26-Apr-2007	##########	2	I21.208	I21.208	2	I25.101	2

2,007	400744236	1	1	1	83	16-Oct-2007	02-Nov-2007	1	I25.105	I21.002	1	E78.501	2
2,007	400686320	1	1	2	71	10-Jul-2007	20-Jul-2007	2	I21.004	I21.004	1	I21.002	01
2,007	400011489	4	7	2	68	05-Feb-2007	12-Feb-2007	2		I21.009	2	I25.103	2
2,007	400688385	1	2	2	66	##########	##########	2	I21.304	I21.403	1	I50.902	2
2,007	400688609	1	1	1	70	04-Jan-2007	17-Jan-2007	2	I21.907	I21.907	2		
2,008	400686312	3	1	1	52	07-Apr-2008	16-Apr-2008	2	I21.207	I21.207	2	I25.105	2
2,008	400688545	4	1	1	65	07-Jan-2008	21-Jan-2008	2	I25.105	I21.001	1	I10 05	2
2,008	400014014	1	1	1	48	##########	##########	3	I21.103	I21.103	2	I25.105	2
2,008	400688385	1	4	2	79	22-Feb-2008	01-Mar-2008	3	I21.403	I21.403	1	I50.902	2
2,008	400688609	1	1	1	66	##########	##########	2	I21.002	I21.002	2	I25.105	2
2,008	400010558	1	1	1	73	03-Feb-2008	18-Feb-2008	2		I21.002	2	I25.105	2
2,009	400686312	1	2	1	79	##########	##########	3	I21.403	I21.403	2	I25.105	2
2,009	400686312	1	3	1	75	24-Jun-2009	10-Jul-2009	2	I21.103	I21.103	2	I25.105	2
2,009	400686312	1	2	1	56	##########	21-Oct-2009	3	I21.103	I21.103	2	I25.105	2
2,009	400688545	3	1	1	44	##########	06-Jan-2009	2	I25.105	I21.403	2	I25.105	2
2,009	400688545	6	1	1	56	14-Nov-2009	21-Nov-2009	2	I25.105	I21.002	2	I25.105	2
2,009	102174925	1	3	1	60	19-Jan-2009	18-Feb-2009	2	I21.002	I21.002	1	I25.105	2
2,009	102174925	1	3	1	77	09-Nov-2009	##########	3	J45.901	I21.103	1	I25.105	2
2,009	400012916	1	1	1	41	28-Oct-2009	02-Nov-2009	1	I21.403	I21.403	2	I50.902	2
2,009	400686347	6	1	1	61	##########	##########	3	I21.103	I21.103	1	I25.105	2
2,009	400744236	1	1	1	60	12-Mar-2009	26-Mar-2009	1	I21.403	I21.403	1	I10 05	2

2,009	400011489	3	1	1	48	26-Nov-2009	##########	3		I21.002	2	I25.105	2
2,009	400688385	3	1	1	35	26-Oct-2009	30-Oct-2009	1	I20.902	I21.002	1	I50.902	2
2,009	400688609	3	1	1	53	11-Mar-2009	18-Mar-2009	2	I21.002	I21.002	2	I25.105	2
2,009	400688609	3	1	1	31	09-Mar-2009	16-Mar-2009	1	I21.002	I21.002	2	I25.105	2
2,009	400010558	1	1	2	82	01-Apr-2009	10-Apr-2009	3		I21.002	2	I25.105	2
2,009	400000886	3	1	1	38	##########	##########	3	I21.002	I21.002	2	I25.105	2
2,009	400000886	3	1	1	58	02-Nov-2009	18-Nov-2009	3	I21.103	I21.103	4	I21.002	4
2,010	400686312	6	1	1	68	##########	##########	2	I21.105	I21.105	2	I25.105	2
2,010	400688545	1	1	1	53	06-Apr-2010	17-Apr-2010	3	R07.401	I21.004	2	I51.903	2
2,010	400688545	3	1	1	65	26-Jul-2010	##########	2	I25.105	I21.002	2	E78.501	2
2,010	400688545	1	1	1	69	08-Jul-2010	14-Jul-2010	3	I21.103	I21.103	2	I10XX03	2
2,010	400686347	1	1	1	82	02-Apr-2010	17-Apr-2010	3	I21.403	I21.403	1	I25.105	2
2,010	400014014	3	1	1	38	18-Jan-2010	22-Jan-2010	2		I21.902	2	I51.903	2
2,010	400688385	1	1	1	57	02-Mar-2010	15-Mar-2010	1	I21.103	I21.103	1	I50.902	1
2,010	400688385	6	1	1	39	08-Jul-2010	18-Jul-2010	2	I25.105	I21.002	1	I50.902	2
2,010	400688609	1	1	1	77	03-Mar-2010	22-Mar-2010	3	I21.103	I21.103	2	I25.105	2
2,010	400000886	6	1	1	73	02-Jul-2010	14-Jul-2010	2	I21.403	I21.403	1	Z95.804	1
2,010	400686291	1	1	1	54	13-Jul-2010	23-Jul-2010	2	I24.803	I21.403	2	I25.105	2

Continued:

S0506	S050601	S0507	S050701	S0508	S050801	S0509	S050901	S0510	S051001	S0511	S051101	S0503	S0504	S050
I50.908	2	J42 02	2	J98.402	2	I51.709	5	I27.001	5	I34.001	5			
I10 05	1	I63.901	2	G45.001	2	K80.203	5							
E11.901	2	E78.502	2											88.5
I51.903	2													
R57.001	2										2	0	0	36.9
I69.301	4													
I48 01	2	I10 02	2	E11.901	2	E78.502	2	I65.205	2					88.5
I50.907	2	I25.206	3	I10 04	2	E78.501	2	E11.901	2	E87.802	2			
I50.902	2	I25.210	2	I10 05	2	E78.501	2							36.0
I50.907	2	I10 02	2											36.0
J98.402	1	N18.905	2	K27.903	2									
I25.101	02	I50.908	02	E78.902	02									36.0
I51.703	2	I50.905	2	I10 21	2	E11.901	2	E78.503	2					
I45.102	5	I42.202	5	I10 04	2	B17.101	5							36.0
I49.301	2	I50.907	3											36.0
E11.901	2	I69.802	5	M06.991	2	K25 01	2							36.0
I51.903	2										2	0	0	0
I10 04	2	E11.901	2	J18.803	1									
I51.903	2	R77.801	2	R73 02	2	J18.901	2					J18.901		88.5
I51.903	5	E11.901	2											89.1
I25.203	2	Z98.8112	5	Z95.804	5									88.5
I50.902	2	I25.208	2	I10 05	2	N18.905	2	E78.501	2	J98.402	1			
I50.902	2	E78.501	2	E77.801	2	J98.402	2					J98.402		36.1
I51.903	2	I10 05	2											36.0
I50.907	2	I25.302	2											36.0
I48 06	1	I49.002	1	I51.709	2	I50.902	2	K80.002	1	J40 03	1			36.0
I48 04	2	I50.908	2	J44.003	2	J15.001	2	J15.101	2	J15.201	2	A04.903		96.0

I10 03	2	E78.501	2	R73 02	2									36.0
I51.903	2	I10 05	2	B18.201	5	I69.301	5	R94.501	2					36.0
E78.501	2													36.0
I51.903	2													36.0
														88.5
I51.903	2	I49.904	2	I49.002	1	R77.801	2							00.4
I51.903	2	I10 04	2	Q61.301	5	R77.801	2							87.4
I51.707	2	I50.908	2	I10 05	2	J98.402	2							88.7
I51.903	2	E78.501	2										0	88.5
I49.904	4	I25.105	4	I50.908	4	E11.901	4	E78.501	4	K76.001	4		0	36.1
E78.501	2	I50.902	2	I10xx04	2									36.0
I10XX04	2													36.0
E79.001	2													36.0
E78.501	2													36.0
N18.905	2	R94.501	1	D64.903	2	E79.001	2	I50.907	2	I70.904	2			
I25.105	2													
I45.102	2	I44.001	2	I44.101	2	I10 05	2	E11.901	2	J06.902	2			36.0
														00.6
I50.907	2	I10 04	2	J42 02	2	K81.101	5	N28.101	5					36.0
E78.501	2	N40xx01	2	I10xx02	9									88.5
I50.907	2	I10xx05	2	R94.501	2	E78.501	2	E79.001	2	I49.804	2			36.0
