# Supplementary material for: Variations in the Quality of Care at Large Public Hospitals in Beijing, China: A Condition-Based Outcome Approach
Source: PLoS One. 2015 Oct 2;10(10):e0138948. doi: 10.1371/journal.pone.0138948 (PMC4592271; doi:10.1371/journal.pone.0138948)
Supplement: S1 Table — (PDF) [file pone.0138948.s004.pdf]

S1_Table

Inclusion and exclusion criteria for case selection

Measure Name	Inclusion Criteria	Exclusion Criteria
Measure #1: Risk-standardized Mortality Rate for AMI patient (RSMR-AMI)	I21	a) age<18 and age>90; b) inpatient stay<1 day; c) transferred to another acute care hospital; d) pregnancy, childbirth, and puerperium conditions (O00-O99); e) missing discharge status
Measure #2: Risk-standardized Mortality Rate for Stroke patient (RSMR-Stroke)	I61-I64	
Measure #3: Risk-standardized Mortality Rate for Pneumonia patient (RSMR-Pneumonia)	J10-J18	
Measure #4: Risk-standardized Mortality Rate for CABG patient (RSMR-CABG)	36.10-36.19	
Measure #5: Risk-standardized Post-surgical Complication Rate (RSCR)	I26.x, I80.2, I44.1, I44.2, I46.x, I47.2, I48.x, I50.x, J90.x, J93.x, N99.0, T81.4, J13.x-J16.x, J19.x, I61.x-I64.x, A40.x, N39.0, R57, T81.1, R58, T80.1, T81.5, K92.2, other T-codes	Also excluded patients with only procedure codes 87-99 (miscellaneous diagnostic and therapeutic procedures)
Measure #6: Risk-standardized Failure-to-Rescue Rate (FTR)		
